# Placing children and adolescents at the centre of the Sustainable Development Goals will deliver for current and future generations

**DOI:** 10.1080/16549716.2019.1670015

**Published:** 2019-10-07

**Authors:** Tobias Alfvén, Johan Dahlstrand, David Humphreys, Daniel Helldén, Sofia Hammarstrand, Anna-Clara Hollander, Mats Målqvist, Sahar Nejat, Peter Søgaard Jørgensen, Peter Friberg, Göran Tomson

**Affiliations:** a Department of Public Health Sciences, Karolinska Institutet, Stockholm, Sweden; b Sachs’ Children and Youth Hospital, South General Hospital, Stockholm, Sweden; c Swedish Institute for Global Health Transformation (SIGHT), Royal Swedish Academy of Sciences, Stockholm, Sweden; d Department of Occupational and Environmental Medicine, Sahlgrenska University Hospital and University of Gothenburg, Gothenburg, Sweden; e Emergency Department, Townsville Hospital and Health Service, Townsville, Australia; f Department of Occupational and Environmental Medicine, Sahlgrenska University Hospital and University of Gothenburg, Gothenburg, Sweden; g Epidemiology of Psychiatric Conditions, Substance use and Social environment (EPICSS), Department of Public Health Sciences, Karolinska Institutet, Stockholm, Sweden; h International Maternal and Child Health, Department of Women’s and Children’s Health, Uppsala University, Uppsala, Sweden; i Paediatric Public Health Department, Sachs’ Children and Youth Hospital, South General Hospital, Stockholm, Sweden; j Stockholm Resilience Centre, Stockholm University, Stockholm, Sweden; k Institute of Medicine, Sahlgrenska Academy, University of Gothenburg, Sweden; l Department of Learning, Informatics, Management and Ethics, Karolinska Institutet, Stockholm, Sweden

**Keywords:** Child health, children, Sustainable Development Goals, multisectoral, health equity

## Abstract

Child health is taking the back seat in development strategies. In summarising a newly released collaborative report, this paper calls for a novel conceptual model where child health takes centre stage in relation to the 2030 Agenda and the Sustainable Development Goals. It lays out five principles by which renewed effort and focus would yield the most benefit for children and adolescents. These include: re-defining global child health in the post-2015 era by placing children and adolescents at the centre of the Sustainable Development Goals; striving for equity; realising the rights of the child to thrive throughout the life-course; facilitating evidence informed policy-making and implementation; and capitalising on interlinkages within the SDGs to galvanise multisectoral action. These five principles offer models that together have the potential of improving design, return and quality of global child health programs while re-energising the 2030 Agenda and the Sustainable Development Goals.

Advancing global child and adolescent health involves much more than achieving gains towards the under-five and neonatal mortality targets enshrined within the United Nation’s 2030 Agenda. Progress should not only be defined by survival of our youngest, but the recognition and realisation of these children’s right to thrive and to lead fulfilling lives throughout the life course [1].

While mortality indicators are essential for monitoring progress, they do not provide a comprehensive picture of the burden of disease borne by children and adolescents, and are a poor benchmark for measuring how best to ensure the wellbeing of the next generation [2]. Children and adolescents need to thrive, empowered with sufficient resilience to withstand looming global challenges such as climate change, armed conflict, forced migration and an evolving burden of non-communicable disease. Moreover, the health and wellbeing of children and adolescents forms the core of what will become society’s future human capital, a resource to create tomorrow’s affluence [3].

Through an interdisciplinary collaboration between the Swedish Institute for Global Health Transformation (SIGHT) and the Swedish Society of Medicine, a road map for global child health in the era of the Sustainable Development Goals (SDGs) has been developed [4].

The initial framework for this originated from a roundtable meeting on how Sweden can best contribute to global child health within the context of the SDGs. The meeting was hosted at the Royal Swedish Academy of Sciences by SIGHT in Stockholm in April 2017. From this a writing group of contributors with backgrounds in paediatrics, public health, psychology, economics, and environmental sciences was formed. Early in the process a review of the vast literature concerning global child health was undertaken, with a particular emphasis on current trends and priorities within the post-2015 era. Continuous meetings and discussion within the writing group facilitated an interdisciplinary approach to the analysis. Moreover, preliminary findings were presented and discussed at both the 2017 European Public Health Conference and the 2018 Swedish Global Health Research Conference as well as in meetings with other stakeholders, providing key input [4].

In calling for a transformative agenda of child-centred development, this road map outlines how exploring interlinkages between child and adolescent health and other sectors, whilst harnessing synergies within the SDGs for children and adolescents (Figure 1), could together rejuvenate efforts and lead to lasting progress [2].

**Figure 1.**
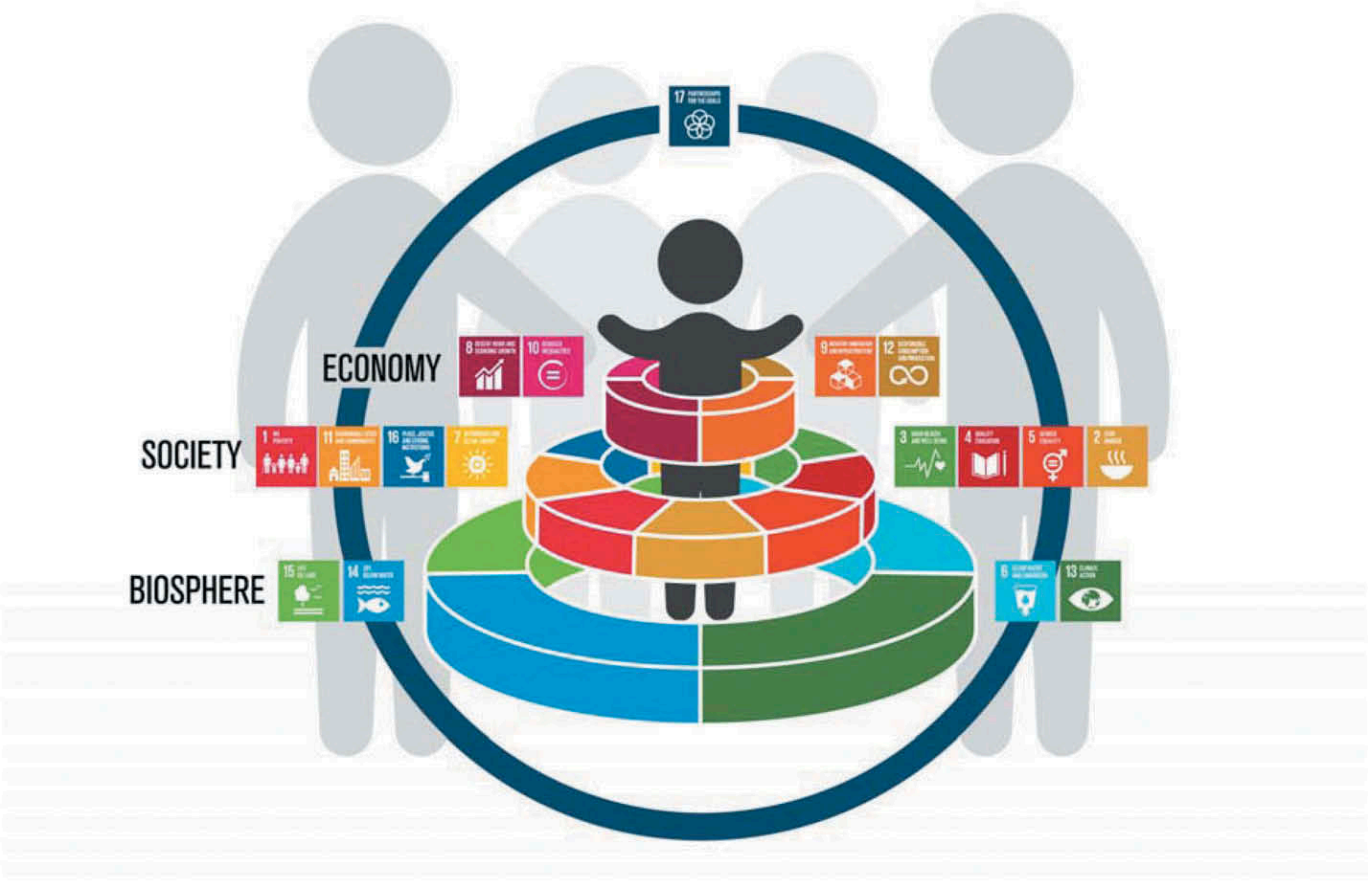
Redefining global child health in the post-2015 era: placing children at the centre of the Sustainable Development Goals.

The road map identifies and focuses attention on five main principles:

1. Redefining global child health in the post-2015 era: placing children at the centre of the SDGs through a life-course perspective

Comprising 17 goals and 169 targets the SDGs present a complex adaptive system, which from a child health perspective highlights challenges and opportunities. Increasing epidemiological diversity risks scattering the development agenda and disrupting advocacy and cooperation efforts, and thus demands renewed efforts to highlight the importance of investing in the health and wellbeing of children. Our narrative offers a new perspective of the SDG framework by recognising children and adolescents as both leaders and beneficiaries of development, symbolised by placing them at the centre of the SDGs [2]. Such a perspective will also promote meaningful inter-sectoral engagement and galvanise much needed public support for the goals.

(2) *Striving for equity: ensuring no child is left behind*

Despite reductions in global infant and child mortality during recent decades, progress has been uneven. A central tenant of the SDGs agenda, reducing inequity is essential to drive efforts to reach and target populations in greatest need. Investment in, and ensuring access to interventions proven to reduce morbidity and mortality must be supported by continuous scientific and contextual evaluation of their impact, including age and sex-aggregated data, to ensure no child or adolescent is left behind [5]. Here strategic leadership and transparent governance and accountability is paramount, especially from national governments.

(3) *Enabling a child’s right to thrive throughout the life-course*

We promote adjusting the perception of the child from a disease-focused to a holistic, relational and child rights perspective. A rights-based approach to child health will guide policies at a national level towards ensuring that children have the best opportunity to not only survive, but also to thrive [6]. Adopting a life-course approach that defines and recognises the specific child rights needs of different age groups is essential.

Such a life-course approach, which considers the long-term effects of physical and social exposures during different life stages on disease risks [7], in line with the determinants of health hypothesis is central to this child-centred perspective within the SDGs [1,8].

(4) *Bridging the ‘know-do gap’: facilitating evidence informed policy-making and implementation*

In spite of rapid growth in knowledge and technological innovation, evidence-based preventive measures and life-saving interventions are often of sub-optimal quality and still fail to reach those who need them most [9]. Projects such as the World Bank-led Disease Control Priorities (DCP3) initiative and WHO’s Evidence-Informed Policy Network (EVIPNet) aim to make cost-effective strategies available to assist governments and policy-makers to prioritise key interventions. Expanding translational research programs and implementation research that engage and build capacity within local communities plays a vital role in combating emerging global health challenges.

(5) *Capitalising on interlinkages within the SDGs to galvanise multisectoral action*

Identifying and capitalising on interconnections within and between the SDGs and their convergence on the health and wellbeing of children and adolescents is fundamental for promoting effective multi-sectoral partnerships that strengthen the sustainability and resilience of health and social systems. Understanding the nature of these interlinkages will be instrumental, as harnessing synergies may open up for possible win-win, or even win-win-win opportunities [10] whilst the limited number of trade-offs [11] demand diplomacy and facilitation. The latter can be illustrated by the traditional, and false, diversion of the economy and the environment, where short term economic focus on gross domestic product growth does not take into account the harmful environmental externalities and subsequent detrimental effects on children´s health and wellbeing [12].

At a time when political instability and competing investment demands place many global health priorities in jeopardy [13,14], it is more important than ever to articulate the interlinked nature of child health. However, by situating child health within the complex framework of the SDGs and engaging with a wider range of stakeholders in all sectors, there is a real risk of inaction on critical child health aspects as responsibilities are shared more broadly. Indeed, we should not forget the Millennium Development Goals era’s success to lower under five mortality. There are still more than five million children that die before their fifth birthday every year, and the successful, most often health sector interventions launched during the last 20 years have to continue and be expanded where needed. A balanced approach to the perspective of health sector activities versus interdisciplinary collaborations is paramount, where both are appraised and utilized appropriately [15].

Acknowledging that basic human rights are still unrealized for millions of children and adolescents globally [16], there is an urgency to reformulate and revitalise the narrative around global child and adolescent health. It is our ambition that the principles laid out here can initiate such a discussion. Placing children and adolescents firmly at the centre of the SDGs will ensure that they realise the right to survive and thrive throughout the life-course, as the true beneficiaries of the 2030 Agenda. Further, we believe that this will re-energise the overall 2030 Agenda.

## Paper context

The holistic framework enshrined in the Sustainable Development Goals provides the opportunity for understanding and appreciating the interlinked nature of the health and wellbeing of children and adolescents. In order to realise this opportunity, a new bold conceptual framework coupled with an equity and life-course focus that can bridge the implementation gap is necessary. This paper provides the foundation for a revitalised discussion on child and adolescent health in the 21st century.
